# Cognitive Reserve and Anxiety Interactions Play a Fundamental Role in the Response to the Stress

**DOI:** 10.3389/fpsyg.2021.673596

**Published:** 2021-09-03

**Authors:** Jose A. García-Moreno, Fernando Cañadas-Pérez, Juan García-García, María D. Roldan-Tapia

**Affiliations:** ^1^CERNEP Research Center, University of Almeria, Almería, Spain; ^2^CEINSAUAL Research Center, University of Almeria, Almería, Spain

**Keywords:** cognitive reserve, anxiety, learning, stress, cortisol, electrodermal activity

## Abstract

The aims of the present study were to assess the possible interaction between Cognitive Reserve (CR) and State Anxiety (SA) on adrenocortical and physiological responses in coping situations. Forty healthy, middle-aged men completed the Cognitive Reserve Scale and the State-Trait Anxiety Inventory. We used an Observational Fear Conditioning (OFC) paradigm in order to assess emotional learning and to induce stress. Electrodermal activity (EDA) and salivary cortisol concentrations were measured throughout the conditions. Our results indicate that those who indicated having higher state anxiety showed a lower capacity for learning the contingency, along with presenting higher salivary cortisol peak response following the observational fear-conditioning paradigm. The most prominent finding was the interaction between cognitive reserve and state anxiety on cortisol response to the post observational fear-conditioning paradigm. Thus, those who showed a high anxiety-state and, at the same time, a high cognitive reserve did not present an increased salivary cortisol response following the observational fear-conditioning paradigm. Given these results, we postulate that the state anxiety reported by participants, reflects emotional activation that hinders the attention needed to process and associate emotional stimuli. However, cognitive reserve has an indirect relation with conditioning, enabling better emotional learning. In this context, cognitive reserve demonstrated a protective effect on hormonal response in coping situations, when reported anxiety or emotional activation were high. These findings suggest that cognitive reserve could be used as a tool to deal with the effects of stressors in life situations, limiting development of the allostatic load.

## Introduction

Cognitive Reserve (CR) is a theoretical construct referring to the discrepancy between observed brain impairment and clinical deficits manifested (Stern, 2002; Barulli and Stern, 2013). Other researchers define the cognitive reserve by emphasizing how it develops through stimulating activities or occupations, whether physical or cognitive, as well as through recreational activities (Jefferon et al., 2011; Cheng, 2016). Different indices have been used to calculate the cognitive reserve scores, such as years of education, occupational complexity, premorbid IQ, and engagement in recreational activities (see Harrison et al., 2015 for a review).

The cognitive reserve has primarily been related to cognitive decline and dementia, where it is reported to have a moderating effect against genetic vulnerability to dementia (Dekhtyar et al., 2019). It must be emphasized, however, that the roles of cognitive reserve are not limited to compensating for cognitive impairment and diseases related to aging (Roldán-Tapia et al., 2012; Lavrencic et al., 2017; Stern and Barulli, 2019). There is plentiful literature on its influence in recovery from traumatic brain injuries (Schneider et al., 2014; Stenberg et al., 2020) and strokes (Shin et al., 2019; Umarova et al., 2019). It also prevents and delays the appearance of cognitive deficits associated with several diseases like multiple sclerosis, Huntington’s and Parkinson’s disease (Farinpour et al., 2003; Soloveva et al., 2018; Ifantopoulou et al., 2019; Santangelo et al., 2019), and other medical conditions (Brickman et al., 2009; Amodio et al., 2017; Feinkohl et al., 2017) and even in delaying the start and decreasing severity in substance abuse disorders (Cutuli et al., 2019).

Currently, possible relationships between cognitive reserve and stress remain to be studied. Stress is defined as experiences that cause feelings of anxiety and frustration due to demands that are greater than one’s ability to successfully cope (McEwen, 2006; Kudielka et al., 2009). In this regard, the concept of allostatic load has been introduced to refer to events of daily life that alter the functioning of physiological and hormonal systems, leading to reactive behavior patterns (McEwen, 2006).

The allostatic load depends on the impact of life experiences in conjunction with lifestyle habits, diet, exercise and/or genetic load. It may underlie the development and expression of behavioral disorders and of several diseases (Arnsten, 2009; Schwabe and Wolf, 2009; Datta and Arnsten, 2019). An understanding of the variables that determine individual differences is vital in protecting against the impact of allostatic load on health.

Within this area of interest, psychological resilience is the most extensively studied construct as a moderator of stress responses. In their meta-analysis, Sisto et al. (2019) conclude that resilience is understood as a dynamic process of adaptation to different life conditions. This should not be understood as a personal attribute, but as an interaction of factors that reflect a history of positive functioning in the face of adverse events. For example, it has been related to stress management both, in field studies (Babanataj et al., 2019) and in experimental studies (Lehrer et al., 2019), demonstrating that people with high resilience show lower cortisol levels in the face of perceived stress. In this line, a low resilience score is related to greater perceived stress, higher intensity in the perception of daily life events, greater symptoms of depression, obsession and compulsion, as well as higher cortisol concentrations (García-León et al., 2019).

In this scenario, cognitive reserve is a construct that has been identified with years of education and with intellectual and cognitive abilities (see Harrison et al., 2015 for a review). It is related to cognitively, physically and socially stimulating experiences and activities. To date, studies on the possible protective effects of cognitive reserve against stress responses or coping with high cognitive demanding events are scarce. However, we did find one study on the moderating role of cognitive reserve on the effects of allostatic loading. Udeh-Momoh et al. (2019) analyzed the effect of the presence of high levels of cortisol as a predictor of the clinical progression of Alzheimer’s Disease (AD), in conjunction with alterations in amyloid-β (Aβ). Hypersecretion of cortisol accelerated the clinical transition to mild cognitive impairment (MCI) and to AD in cognitively healthy individuals with pathological presence of Aβ42. The cognitive reserve seemed to have a moderating effect on this pathological progression, since high-risk individuals who had high scores in reserve were more likely to show a slower progression of dementia.

To the best of our knowledge, there are no studies that explore a potential effect of cognitive reserve in contexts of coping with stress. In this way, the cognitive reserve could play a protective role not only in the relationship between allostatic load and expression of a pathology, as stated by Udeh-Momoh et al. (2019), but also in the hormonal response pattern to stressful events. The hormonal response is measured by cortisol levels and depends on the activity of the hypothalamic-pituitary-adrenal axis (Liyanarachchi et al., 2017).

Besides that, cognitive reserve would facilitate a response pattern implying less hormonal reactivity, and therefore, with less allostatic load, thereby minimizing the negative effects of stress on cellular aging and neuronal plasticity (McEwen et al., 2015a).

In the experimental context, there are paradigms that simulate conditions like these stressful experiences of daily life (Dickerson and Kemeny, 2004). Particularly notable are studies of observed fear conditioning (OFC) (Olsson et al., 2007; Olsson and Phelps, 2007; Haaker et al., 2017), whether vicarious or direct in nature.

The OFC assesses the ability to learn in an emotional cues’ context through observation. Observation of emotional distress in peers produces sympathetic activation of increased arousal (Vaughan and Lanzetta, 1980; Levenson and Ruef, 1992). This increase is quantified by electrodermal activity (EDA). This is the main physiological response dependent on the activity of the sympathetic system. One way to study electrodermal activity is by analyzing the skin conductance responses (SCRs), the measure most used to assess fear conditioning (Lonsdorf et al., 2017). During OFC, an anticipatory expectancy of a possible aversive event, i.e., a cutaneous electric shock, is induced. Therefore, this observational fear-conditioning paradigm, assessing emotional learning, is a reliable candidate to produce stress responses in participants.

Studies have indicated that anxiety affects attentional capacity and learning (Helsen et al., 2011; Bilodeau-Houle et al., 2020), as well as stress responses in terms of cortisol levels. Fiksdal et al. (2018) study, for example, showed that anxiety levels increased hypothalamic-pituitary-adrenal axis reactivity to social stress. In this direction, Budnick et al. (2019) observed that socially anxious participants showed higher cortisol, following a job interview. However, some studies have obtained contradictory results (Elnazer and Baldwin, 2014; Klumbies et al., 2014; Espín et al., 2016). It is known that HPA axis reactivity is moderated by different variables such as gender, different psychiatric disorders or cross-cultural differences (Zorn et al., 2017; Miller and Kirschbaum, 2018).

Regarding the relationship between anxiety and learning, Moran (2016) reports a negative relationship between anxiety and working memory and executive-attentional processes. Anxiety also affects the learning of observational fear conditioning, Kelly and Forsyth (2007) report that individuals with high levels of anxiety tend to show greater anticipatory behavior, and therefore a poorer ability to discriminate between safe and threatening stimuli. Different publications (Grillon, 2002; Lissek et al., 2005, 2014; Gazendam et al., 2013) indicate that anxious individuals are characterized by increased threat responses to safe stimuli. Although this result is controversial, Selbing and Olsson (2019) indicated that subjects with higher anxiety show a robust discriminative learning.

In order to contribute in this direction, our study investigates the effect of cognitive reserve and the role of anxiety in emotional learning. In this line, we suggest that cognitive reserve plays a protective role in the hormonal responses to situations of emotional learning and coping with stress in the experimental context; to verify this, we selected the observational fear conditioning protocol (Haaker et al., 2017) as an emotional learning and stress-inducing paradigm.

## Materials and Methods

### Participants

A total of forty-five healthy, middle-aged men (M = 50.5 years old, SD = 8.6, range 35–65) voluntarily participated in the study. Participants were recruited via an e-mail announcement sent to the university community of the University, the Spanish Agency of Research and the School of Music of Almeria (Spain), as well as through snowball sampling. The inclusion criteria were as follows: male, between the ages of 35-65 years, living in Almería and having Spanish nationality. The exclusion criteria were: (i) a history of psychiatric, neurodegenerative or endocrine disease, (ii) substance addiction or abuse, (iii) existence of a severe or unstable medical condition, (iv) current use of psychiatric medication or medication that may alter cognitive states, (v) altered circadian rhythm due to factors such as shift work or an altered sleep schedule.

The men who took part in this study are of Spanish nationality, residents of Almeria, with heterogeneous academic backgrounds and professions such as university professor, researchers, farm workers, accountants, businessmen, bricklayers, etc.

As compensation for the participation in the study, each participant received a customized report according to their scores in cognitive reserve, in state/trait anxiety and their cortisol levels. Two participants were excluded from the data analysis because they expressed suspicion about whether shocks were being administered during the study. Three participants were excluded due to technical problems. Forty participants remained in the final analysis (see Table 1).

**TABLE 1:** Demographic and other descriptive variables.

		Cognitive reserve		
	
	Low		High	
		
	M	SD	M	SD
Age	51.3	7.25	49.7	10.0
Cortisol base line	6.0	3.94	6.9	3.47
Cortisol peak response	14.8	6.54	8.9	4.73
Cortisol recovery	7.6	4.74	7.17	4.57
State anxiety	51.6	9.78	45.7	9.07
Trait anxiety	41.3	11.8	37.9	9.11

The study was reviewed and approved by the University of Almería Bioethics Committee (UALBIO2020/026) and carried out according to the Declaration of Helsinki.

### Instruments

#### State-Trait Anxiety Inventory

The State-Trait Anxiety Inventory (STAI) (Spielberger, 1989) in the Spanish version (Buela-Casal et al., 2016) was used. This questionnaire assesses (1) state anxiety (SA), defined as a transitory emotional state, characterized by consciously perceived, subjective feelings of attention and apprehension, and by hyperactivity of the autonomic nervous system; and (2) trait anxiety (TA) as a relatively stable propensity to anxiety, characterizing individuals with a tendency to perceive situations as threatening. The inventory has shown high reliability STAI reliability with an alpha value between 0.87 and 0.93 (Guillén-Riquelme and Buela-Casal, 2014). In the present study, the statistic alpha was = 0.88. The scores in the state-anxiety subscale were divided into two levels (High and Low), based on the median, in order to evaluate its potential effects on learning and on neuroendocrine response.

#### Cognitive Reserve Scale

The Cognitive Reserve Scale (CRS) (León et al., 2011) was used. This scale provides an index that indicates participation in cognitive stimulation activities such as reading, playing musical instruments, hobbies like collecting trading cards, speaking different languages, traveling, doing sports, etc. The CRS uses a Likert-type scale from 0 to 4 for response to 24 items. The scale has shown high reliability (alpha = 0.81) and adequate content validity. In the present study, the statistic alpha was = 0.80. The total score is the sum of scores from all the items. The scores in the cognitive reserve scale were divided into two levels (High and Low), based on the median, in order to evaluate its potential effects on neuroendocrine response.

### Stress Induction Paradigm

Our procedure was adapted from Olsson and Phelps (2007). The observational fear conditioning protocol consists of two phases: The first phase, or vicarius, in which the participant observes a video in which a male model is shown with two electrodes attached to his right forearm, in the same experimental room as the participants, with identical stimuli. On its screen (14-inch), one of two colored squares (blue and yellow) were shown, serving as conditioned stimuli (CS). The different CS were presented during 10 s in pseudo-random order. A 10-s intertrial interval (ITI) was established between the different CS. During the ITI, the word *descansa* [rest] was displayed on the screen. Each colored square was presented ten times, beginning with a rest period. The colors were presented in a pseudo-random sequence, never allowing more than two CS+ or CS− stimuli to be presented successively. Seven of the presentations of the color that acted as CS+ were contingent with the actor’s simulation of getting an unpleasant electric shock to his right wrist, while the other color (CS−) was never paired with the shock (Unconditioned Stimulus, US). The first, sixth and ninth CS (CS + 1, CS + 6 and CS + 9, respectively) were not associated with the US. We did not counter-balance the color used as CS+; the same color was selected for all participants (blue square). The second phase, direct phase, two electrodes were attached to the participant right forearm, in the same experimental room as during de previous phase. Another video was designed (6 min, 00 s) like the video used during the vicarious phase. The same one of two-colored squares (blue and yellow) were shown in a pseudo-randomized order and again never allowing more than two CS+ or CS− stimuli to be presented successively. In this phase the CS were never reinforced with an electric shock. In both phases, we use the Biotrace + Nexus 10 software (MindMedia, 2017) to mark the onset of the CS+, CS−, and US (the last just during the vicarious phase).

### Skin Conductance Response

The SCR was registered during the OFC protocol as an index of learning. For analysis of the SCR we register the electrodermal activity (EDA) following the guidelines set by Olsson et al. (2007). Skin conductance responses was determinated for each stimulus onset as the greatest amplitude difference (μS) from base to peak in skin conductance, during a latency window of 0.5 to 4.5 s after the start of the stimulus. The minimum response criterion was 0.02 μS; responses that did not meet this criterion were recorded as “0.” Low pass filtering was applied to the data and distributions were normalized by square root transformation (Lykken and Venables, 1971).

Electrodermal activity (EDA) was measured by Ag–AgCl electrodes placed on the distal phalanges of the second and fourth fingers of the left hand, using an EDA sensor (Nexus-10MKII, Mind Media, Netherlands). The EDA signal was amplified and recorded using NEXUS Systems (Mind Media BV, Roermond-Herten, Netherlands) and its skin conductance module connected to an HP Pavilion dv6 computer, with BioTrace + Software. Data was continuously recorded at an acquisition rate of 265 Hz (samples per second). Pre-processing of the signal was carried out off-line, to extract its analog waveforms, specifically, its phasic skin conductance response to the stimuli from the OFC paradigm. This was performed using the R 3.5.1 software (The R Foundation for Statistical Computing, Vienna, Austria).

### Cortisol Analysis

Salivary cortisol samples were obtained by deposition in an Eppendorf. Salivary cortisol concentrations were analyzed using an ELISA test kit for cortisol in saliva (Labor Diagnostika Nord, Nordhorn, Germany) and quantified by spectrophotometry (Beckman Coulter, Inmunotech, United States). All the samples were analyzed in duplicate and the results from each of the samples are expressed in nmol/L, following the manufacturer’s instructions. All samples were assayed in duplicate, with intra- and inter-assay CV’s below 10%.

### Procedure

**FIGURE 1:**
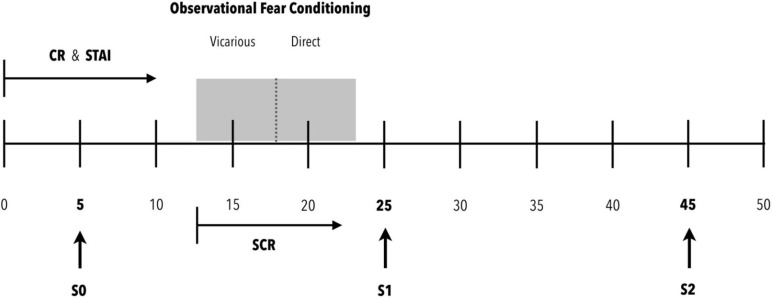
Procedure. Participants arrive at the experimental room (0 min). They are provided with the Cognitive Reserve (CR) and State-Trait Anxiety (STAI) scales for completion. Within 5 min of arriving in the experimental room, the first saliva sample (S0) is requested to establish the baseline cortisol concentration. Ten minutes after arriving in the experimental room, once the scales have been completed, the observational fear conditioning protocol is initiated. For this purpose, the electrodes for recording the electrical conductance of the skin are placed and the appropriate checks are made to ensure a correct recording of the signal. After these actions, the observational fear conditioning protocol is initiated. This protocol consists of two phases: vicarious and direct. During both phases, the electrical skin conductance (SCR) is recorded. At the end of the protocol, the skin conductance recording session is saved, and the electrodes are removed from the participant’s hand. After this, the second saliva sample (S1) is obtained. This sample expects to be able to detect peak cortisol concentrations from the beginning of the evaluation protocol (0 min). After 20 min of rest, the third saliva sample (S2) is obtained to evaluate a possible recovery toward basal cortisol levels.

Participants were assessed individually on a fixed day of the week (in our case, Wednesdays), between 11 and 2pm, at a rate of 5 participants per session. [*T.N. The Spanish typically break for their midday meal at 2 or 3pm.*] In this way, we sought to maximize the similarity of assessment conditions for all participants, avoiding differences in their neuroendocrine responses caused by different circadian rhythms and the light/darkness cycle (Adam et al., 2017). Three saliva samples were obtained at specific times: before starting (S0, baseline), after finishing the OFC protocol (S1, cortisol response) and 20 min after the end of the OFC protocol (S2, cortisol response) (see Figure 1 for all the procedure details). In order to avoid individual differences from different circadian rhythms, participants were asked to obtain a saliva sample at 8am the day prior to the experimental session day. Moreover, participants were encouraged to try to get up each day at 8am during the two weeks prior to the assessment. In addition, they were requested to come to the session while fasting, with proper oral hygiene using plenty of water. The wake-up hour as well as the saliva collection time were registered.

Upon arrival at the laboratory, the participants were placed in a quiet room, S0 saliva sample was obtained and completed a booklet containing the CRS and STAI questionnaires. The OFC was carried out using an laptop with a 14-inch screen. Participant were informed about the possibility of stopping the experiment in anytime. At the beginning of the vicarious phase, participants are informed that they will watch a video of a person performing an experiment similar to the one they have to perform afterward. The instructions indicated in the Olsson and Phelps (2007) protocol were provided.

Once the video was ended, participants were informed that they would be presented the same stimulus watching previously, but in a different order. They were informed that they would receive shocks associated with the same color as the model, with the difference that the person in the video received seven shocks, while they would receive between one and three (that is, at least one and no more than three). They were told that there would be no shocks associated with the other color, nor during rest periods. The objective was to maintain unpredictability and uncontrollability until the last CS + was presented. Importantly, no shock was administered. Before starting this second direct phase, the instructions indicated in the Olsson and Phelps (2007) protocol were provided.

Once the OFC protocol was ended, the second saliva sample (S1) was obtained. This sample was expected to detect peak cortisol concentrations from the beginning of the evaluation protocol (0 min). After 20 min of rest, once the conditioning protocol was ended, the third saliva sample (S2) was obtained to evaluate a possible recovery toward basal cortisol levels. The samples were stored at 4°C and them were stored at −20°C until their later analysis. After thawing, each sample was centrifuged for 10 min at 1500 “× g” in order to remove the pellet and collect the supernatant (Cheetham-Blake et al., 2019). The sample was diluted with standard A in a 1:10 ratio, following the protocol instructions included in the kit.

All participants were informed of the general purpose of the experiment and the methods involved, without revealing information about the contingencies of the experiment or the working hypothesis, and they gave their written informed consent.

### Data Analysis

Data were analyzed in the present study by mixed analysis of variance (ANOVA) or split-plot. A total of three mixed ANOVAs were carried out to investigate the effect of CR and SA on learning capacity and on pattern of stress coping.

For the vicarious phase, five SCR averages were selected (positive conditioned stimulus, CS+; negative conditioned stimulus, CS−; unconditioned stimulus, US). The first two CS+ are not predictive of the US, since the association between the CS+ and the US is first introduced in the second CS+. Hence, the first CS+ that is predictive of the US would be the third one. Considering this, the first two conditioned stimuli (both positive and negative) were averaged to obtain the electrical conductance responses (SCRs) to the neutral stimuli, i.e., before the unconditioned stimulus is first presented. These scores were referred to as CS + PRE and CS-PRE, respectively. The remaining eight conditioned stimuli (both positive and negative) were averaged to obtain the SCRs to the conditioned stimuli once the US has been presented. These scores were referred to as CS + POST and CS-POST, respectively. Therefore, in the vicarious phase of the fear conditioning paradigm the CS + and CS− SCR average responses are distinguished as either before (pre-conditioned stage, PRE) or after (post-conditioned stage, POST) the US was presented. The fifth measure of average SCRs is the one referring to the seven presented unconditioned stimuli.

Using the averages from the last eight CS+, two conditioning groups were established from the vicarious phase (High Conditioning – COND-High – and Low Conditioning – COND-Low −), based on the median. In the direct phase, two average scores were obtained (CS+ and CS−) for each participant. In this manner, we confirm whether the participants who were high conditioned during the vicarious phase have a greater SCR average response to CS + during the direct phase.

Two dependent variables were used: first, the electrical skin conductance responses as an index of learning with contingencies CS-US, and the phasic concentrations of salivary cortisol (S1, cortisol response) as an indicator of the stress response. In cases where the sphericity assumption is not met, the correction of degrees of freedom is estimated using the Huynh-Feldt approximation.

First, to verify the effect of state-anxiety on conditioning during the vicarious phase, we carried out a 5 × 2 mixed ANOVA having one within-group factor (stimuli) with five levels (CS-PRE, CS + PRE, CS-POST, CS + POST, US) and one between-group factor (SA) with two levels (SA-High and SA-Low). Second, to check whether the vicarious conditioning was transferred to the conditioned response during the direct phase, we carried out a 10 × 2 × 2 mixed ANOVA having one within-group factor (CS phases) with ten levels, one within-group factor (US predictor) with two levels (CS+ and CS−) and one between-group factor (vicarious conditioning) with two levels (COND-Low and COND-High). The ten levels of CS presentation were introduced in order to be able to study possible habituation to the conditioned response. Third, to verify the effect of cognitive reserve and state-anxiety on the neuroendocrine phasic response (cortisol response), we carried out a 3 × 2 × 2 mixed ANOVA having one within-group factor (phases) with three levels (baseline, cortisol response and recovery), one between-group factor (SA) with two levels (SA-High and SA-Low), and one between-group factor (CR) with two levels (CR-High and CR-Low).

Effect size was calculated using partial eta squared or Cohen’s *d*, depending on the case. Effect size was interpreted as small (η^2^_*p*_ = 0.01; *d* = 0.20), moderate (η^2^_*p*_ = 0.06; *d* = 0.50) or large (η^2^_*p*_ = 0.14; *d* = 0.80), following Cohen’s recommendations (Cohen, 1988, 1992).

Two complementary analyses were carried out in order to better understand the relationship between three of the study variables, namely, cognitive reserve, state anxiety and conditioning. On the one hand, we carried out an analysis of bivariate and partial correlations between the variables in order to find the degree and direction of the relationships, as well as the relationship between each pairing of variables, controlling for or equalizing the influence of the third. On the other hand, a linear regression analysis was carried out between the CS+ responses during the vicarious phase and during the direct phase, in order to find the degree of transfer from vicarious learning of the CS-US contingency to the conditioned response during the direct phase.

The results with *p* < 0.05 were considered statistically significant. The data analyses were performed with IBM SPSS Statistics for Mac, version 25 (IBM Corp., Armonk, N.Y., United States). In addition, a power sensitivity analysis was performed using G^∗^Power 3.1.

## Results

First, no interaction or statistically significant direct effect of anxiety and/or cognitive reserve was found in the saliva sample obtained the day before the evaluation.

### Does State-Anxiety Affect Vicarious Learning?

**FIGURE 2:**
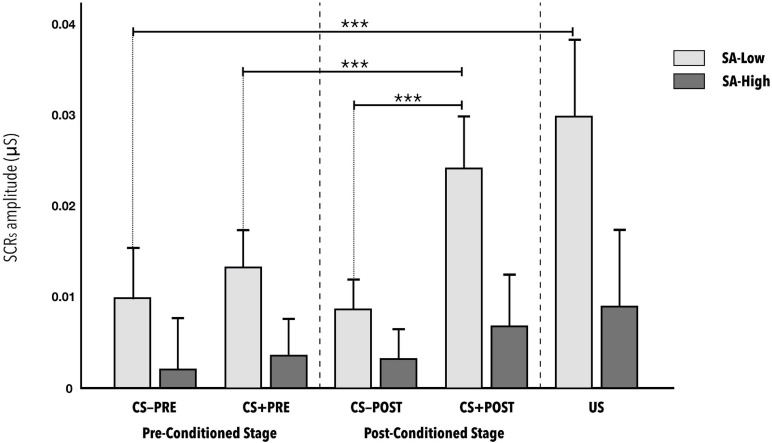
Mean amplitudes of skin conductance responses to the CS−, CS+, and US stimuli during the vicarious phase of the fear conditioning paradigm, according to reported level of state anxiety. CS− and CS+ responses are distinguished as either before (pre-conditioned stage) or after (post-conditioned stage) the US was presented. No statistically significant differences were observed between CS-PRE vs. US (*d* = 0.747), between CS + PRE vs. CS + POST (*d* = 0.639), or between CS-POST vs. CS + POST (*d* = 1.490), indicating correct conditioning in the SA-Low group. The error bars show standard error. Significance levels *p* < 0.001 are represented with ***.

Second, the results for estimating the effect of state-anxiety on conditioning during the vicarious phase that is, on learning the CS-US contingency expressed as the average amplitude of the skin conductance response shown a statistically significant interaction of state-anxiety (SA) with the different stimuli presented (SA^∗^Stimuli) [F_(3__.127__, 118__.8__)_ = 3.933, *p* < 0.01, η^2^_*p*_ = 0.09; 1- β power = 0.9] (see Figure 2). The effect size can be considered as medium. The minimum effect size detectable estimate by power sensitivity analysis was η^2^_*p*_ = 0,06.

The *post hoc analyses* using the Bonferroni correction indicate statistically significant differences in the skin conductance response of the SA-Low group to the different stimuli in the conditions: CS-PRE *versus* US (*d* = 0.747); CS + PRE *versus* CS + POST (*d* = 0.639); CS + PRE *versus* US (*d* = 0.736); CS-POST *versus* CS + POST (*d* = 0.991). Significant differences were also indicated in the CS + POST stimuli (*d* = 1.490) and US (*d* = 0.917), depending on state-anxiety level (see Table 2). No other relevant, statistically significant effects were found in the rest of the *post hoc* combinations in the SA-High group or in any of the combinations in the SA-Low group. Note the absence of significant differences in the initial responses to the CS-PRE *versus* CS + PRE, whether in the SA-Low group or in the SA-High group. All the *p-values* of the *post hoc* analyses indicated as statistically significant in this ANOVA are <0.01.

**TABLE 2:** General *post hoc* table of the different mixed ANOVAs.

*Post hoc*			*p* values	d-n
Low state anxiety (SA-L)	CS-PRE vs. US		0.001	0.747
	CS + PREvs.US		0.001	0.736
	CS + PRE vs. CS + POST		0.006	0.639
	CS-POST vs. CS + POST		<0.001	0.991
CS + POST	SA-Low vs. SA-High		<0.001	1.490
Unconditioned Estimulus (US)	SA-Low vs. SA-High		0.001	0.917
High Conditioned Group (COND-H)	CS− vs. CS+	1–6 7	<0.001	> 0.543
			0.115	0.352
		8	0.082	0.473
		9	0.154	0.255
		10	0.204	0.209
CS + Direct	COND-L vs. COND-H	1–6	<0.001	> 0.882
		7	0.055	0.626
		8	0.675	0.134
		9	0.512	0.209
		10	0.275	0.350
	COND-H (CS + 1 vs. CS + 5}		0.002	0.646
Cortisol peak	SA-Low vs. SA-High	CR-L	<0.001	2.599
		CR-H	<0.05	0.832
	CR-L vs. CR-H	SA-H	<0.001	1.856
CR-L	Baseline vs. Peak	SA-L	<0.001	3.523
		SA-H	<0.001	3.535
CR-H		SA-H	<0.001	1.391

*The p-values are indicated along with distances normalized by Cohen’s *d* to estimate effect size. For the intergroup measurements, d = |μ_*A*_ – μ_*B*_| /root [(n_*A*_ S^2^_*A*_ + n_*B*_ S^2^_*B*_)/(n_*A*_ + n_*B*_)], while for intragroup measurements, the approximation d = t/root(n) was used.*

### Is There a Transfer From Vicarious Learning to Direct Experience?

**FIGURE 3:**
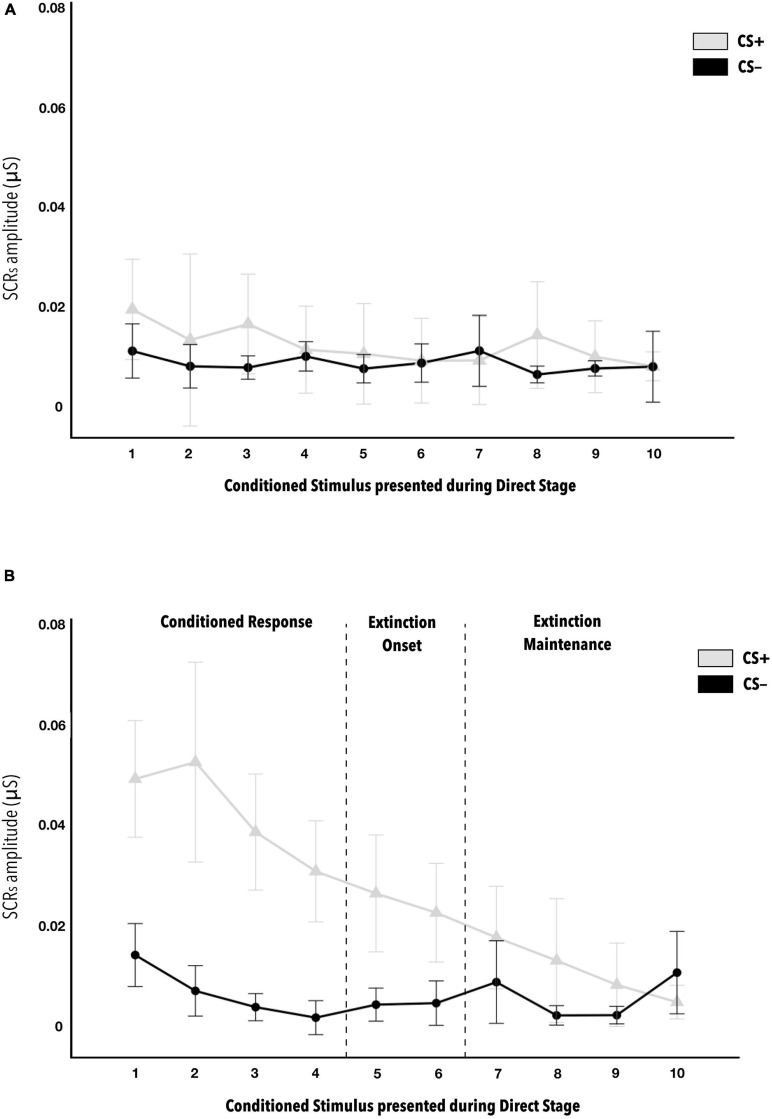
Mean amplitudes of skin conductance responses to CS− and CS+ stimuli during the direct phase of the fear conditioning paradigm, according to level of contingency learning. Figure **(A)** is for the COND-Low group, that is, those that did not show correct vicarious conditioning, while Figure **(B)** is for the COND-H group. In Figure **(B)** of the COND-High group, statistically significant differences are indicated between the first six CS+ *versus* CS− (*d* = 0.563). Beginning with seventh CS+ presentation, no differences were observed with respect to CS−. This indicates when habituation is produced. Statistically significant differences were also observed between CS + 1 *versus* CS + 5 (*d* = 0.646), indicating the start of this habituation. The error bars show standard error. The *post hoc* analyses have *p* < 0.001.

Third, when checking whether vicarious conditioning was expressed during the direct phase, that is, whether those who showed a greater average amplitude of skin conductance response to the eight CS+ stimuli predictive of US during the vicarious phase (COND-High group) would show a greater response to the CS+ versus CS− during the direct phase, the results indicated a statistically significant three-fold interaction (COND ^∗^ US-predictor ^∗^ CS-phases) [F_(__5__.426_, _206__.172__)_ = 3.195, *p* = 0.007, η^2^_*p*_ = 0.08; 1-β power = 0.9]. The US-predictor^∗^CS-phases interaction differed according to the conditioning (COND) group. Thus, the skin conductance response to CS+ and CS− stimuli across the ten presentation phases are different in the COND-Low *versus* COND-High groups (see Figure 3). The effect size can be considered as medium. The minimum effect size detectable estimate by power sensitivity analysis was η^2^_*p*_ = 0.05.

The *post hoc* analyses indicate the absence of statistically significant differences in the COND-Low group for both the US-predictor factor and the CS-phases factor (see Figure 3A). By contrast, statistically significant differences were indicated in the COND-High group between skin conductance response average amplitudes to the first six CS + stimuli and the first six CS− during the direct phase (all *d* ≥ 0.563) (see Figure 3B). This reveals when habituation of the CS+ response was produced. Regarding the CS+, significantly greater average amplitudes were observed in group COND-High *versus* COND-Low in the first six presentations of CS+ in the direct phase (all *d* ≥ 0.882) (see Table 2). This also reinforces the idea of correct transfer of vicarious conditioning to anticipating a contingency directly. Finally, in the COND-Low group there was no difference as a function of USpredictor or CSphases. By contrast, in the COND-High group, significant differences were observed in the skin conductance responses to CS+ between some of their successive presentations, specifically, we note the difference between CS + 1 and CS + 5 (*d* = 0.646), indicating the onset of habituation to CS+ in the COND-High group during the direct phase. There were no important significant differences in CS− as a function of Conditioning or Stimulus factors. All the *p-values* of the *post hoc* analyses indicated as statistically significant in this ANOVA are <0.01.

**FIGURE 4:**
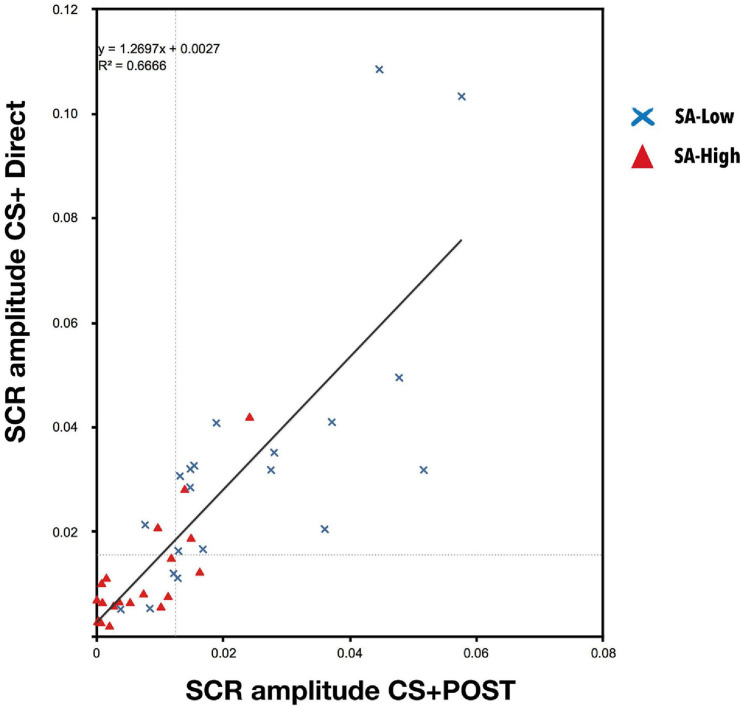
Linear regression between the skin conductance responses to CS + during the vicarious phase (post-conditioned stage, POST) and the skin conductance responses to CS + during the direct phase [*r*^2^ = 0.67, *F*_(1, 38)_ = 77.304; *p* < 0.001]. A high predictive capacity of the vicarious conditioning on the conditioned response was observed during the direct phase, together with a greater response during the direct phase because the US becomes the anticipation of a possible aversive contingency [β = 1.27; *t*_(38)_ = 8.792; *p* < 0.001]. This reinforces the idea of learning transfer.

Linear regression analysis between the average skin conductance responses to CS + POST during the vicarious phase, and the average skin conductance responses of the first six CS+ stimuli during the direct phase, indicated that the participants’ individual skin conductance response amplitude to CS+ during the vicarious phase predicted their responses to CS+ during the direct phase [*r*^2^ = 0.67, *F*_(1, 38)_ = 77.304; *p* < 0.001], reinforcing the idea of conditioning transfer. Moreover, we can observe that the skin conductance responses to CS + during the direct phase are greater than during the vicarious phase [β = 1.27; *t*_(38)_ = 8.792; *p* < 0.001] (see Figure 4).

On the other hand, correlations analysis between three of the study variables (cognitive reserve, state-anxiety, and conditioning), to verify whether cognitive reserve had any effect on CS-US contingency learning, indicated a statistically significant positive correlation of cognitive reserve with the skin conductance response mean amplitude to CS + POST from the vicarious phase (*r* = 0.38, *p* < 0.05) and a negative correlation with state-anxiety (*r* = −0.27, *p* > 0.1). On the other hand, state-anxiety had a statistically significant negative correlation with the skin conductance responses mean amplitude to CS + POST from the vicarious phase (*r* = −0.65, *p* < 0.001). To better understand the role of cognitive reserve, a partial correlations analysis was conducted. First, we obtained a statistically significant partial correlation between state-anxiety and conditioning (*r*_*p*_ = −0.67, *p* < 0.001), with cognitive reserve as control variable. Second, we must note the statistically non-significant partial correlation (*r*_*p*_ = 0.28, *p* < 0.1) between cognitive reserve and conditioning, with state-anxiety as control variable. This indicates an relation between conditioning and state-anxiety. However, if we equalize state-anxiety, we observe no relation between cognitive reserve and conditioning. Consequently, these partial correlations suggest that cognitive reserve has an indirect relation with conditioning through its relationship with state-anxiety.

### Do Anxiety-State and/or Cognitive Reserve Affect the Cortisol Stress Response?

**FIGURE 5:**
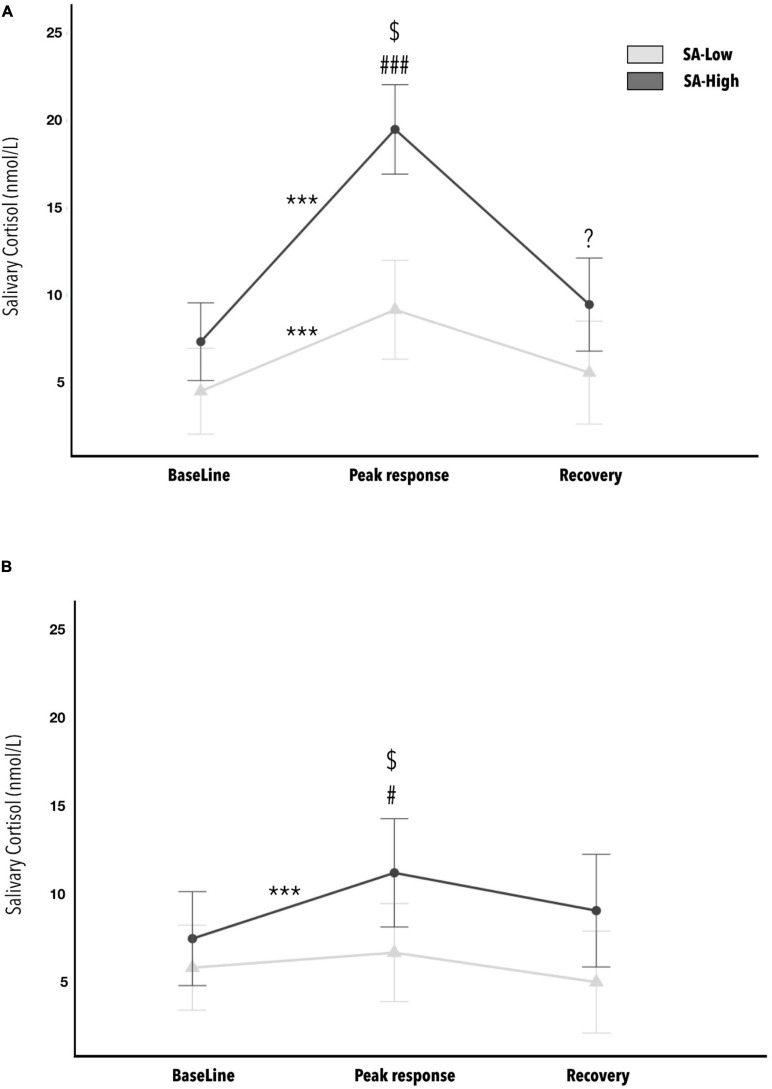
Average concentrations of cortisol in saliva: before (baseline), after (cortisol response) and 20 min following (recovery) the end of the stress-inducing experimental paradigm, as a function of state-anxiety and cognitive reserve levels (Low *versus* High). Figure **(A)** is the CR-Low group, while Figure **(B)** is for the CR-High group. Statistically significant *post hoc* analyses are marked with (*) for comparisons between phases (baseline, cortisol response and recovery). The symbol (#) indicates statistically significant *post hoc* between SA-Low *versus* SA-High groups. The ($) symbol indicates statistically significant *post hoc* with *p* < 0.001 within the SA-High group, between the groups CR-Low **(A)** and CR-High **(B)**. The *p-values* showing one, two, or three * or # represent values <0.05, <0.01, and <0.001, respectively. The error bars show standard error. (*) This symbol refers to within-group comparisons. While this (^#^) symbol refers to between-group comparisons.

Finally, the results for estimating the effect of cognitive reserve and state-anxiety on the neuroendocrine phasic response (cortisol response), due to application of observational fear conditioning, indicated a statistically significant three-fold interaction (CR^∗^SA^∗^Phases) [*F*_(2, 72)_ = 8.530, *p* < 0.001, η^2^_*p*_ = 0.19; 1- β power = 0.96]. The SA^∗^Phases interaction is different for the Low *versus* High cognitive reserve groups (see Figure 5). The effect size can be considered as large. The minimum effect size detectable estimate by power sensitivity analysis was η^2^_*p*_ = 0.11.

The *post hoc* analyses indicated the absence of initial differences due to state-anxiety in cortisol levels prior to the start of the paradigm (S0, baseline), both in the CR-Low group and in the CR-High group. However, in post-paradigm cortisol levels (S1, cortisol response), statistically significant differences were observed between the means of the state-anxiety groups (Low *versus* High), in both the CR-Low group (*d* = 2.599) and the CR-High group (*d* = 0.832). Worth mentioning is a tendency toward significance (*p* = 0.054) between the SA-Low and SA-High groups in the recovery sample (S2), within the CR-Low group. Additionally, in the CR-Low group, statistically significant differences were observed between baseline and cortisol response measurements per the paradigm, for both the SA-Low group (*d* = 3.523) and the SA-High group (*d* = 3.535). On the other hand, in the CR-High group, statistically significant differences were only observed between baseline and cortisol responses measurements in the SA-High group (*d* = 1.391). Finally, within the SA-High group, we note the statistically significant difference between the CR-Low and CR-High groups (*d* = 1.856) in cortisol responses following the observational fear conditioning paradigm. No other relevant statistically significant effects were found in the rest of the *post hoc* combinations. All the *p-values* of the *post hoc* analyses indicated as statistically significant in this ANOVA are <0.05.

## Discussion

In the present study, we have tested a possible interaction between CR and SA in an emotional learning protocol as a stress induction paradigm. This interaction has been assessed through studying cortisol level. Our results indicate a protective effect of the cognitive reserve that reduces levels of stress in participants with high levels of reserve. This effect is mainly showed in participants with high state-anxiety at the beginning of the experiment. Likewise, cognitive reserve was observed to have an indirect relation with emotional learning through anxiety. It has been assessed through studying electrical SCR.

Stress can hinder the capacity to learn. There have been reports of a negative effect of the allostatic load due to stress on the arousal system and attentional processes. This effect may be mediated by the role of the amygdala. In this sense, different studies show that the amygdala and its connection with the prefrontal cortex as the main region of interest in coping with stress (LaBar et al., 1998; Phelps and LeDoux, 2005; McEwen, 2006). It has a central role in learning fear and its relationship with different emotional activation nuclei such as periaqueductal gray matter, the paraventricular nucleus of the hypothalamus for the release of glucocorticoids, the lateral nucleus of the hypothalamus associated with the sympathetic system, and various nuclei of the brain stem. Thus, its functioning affects stress responses as well as attentional processes that are key to learning emotional cues (Davis, 1992; LaBar et al., 1998; Phelps and LeDoux, 2005; Shansky et al., 2010; Barry et al., 2017; Farooqi et al., 2018).

Our results suggest that high emotional activation (high levels of state anxiety), has a negative effect on emotional learning process. This affirmation is limited to the context of coping with observational fear conditioning as an emotional learning and a stress-inducing paradigm. In this regard, one possible explanation is that high emotional activation affects learning by altering attentional processes that make the association possible (Moran, 2016; Shi et al., 2019). This is understood by the lower mean amplitude of skin conductance response, generalized to all stimuli, on the part of participants with high state anxiety.

These results are in line with the theory of attentional control (ACT; Eysenck et al., 2007), which postulates that individuals with high trait anxiety show deficits in executive and attentional processes associated with working memory. These deficits are reflected in poorer performance on cognitively demanding neuropsychological tasks (Berggren and Derakshan, 2013). Several studies (Mogg and Bradley, 2016, 2018; Mogg et al., 2017) claim that such executive and attentional deficits underlie the development and consolidation of anxiety disorders. These deficits can be trained and, in order to do this applied cognitive models of anxiety that propose attention focused on the threat as the cause of anxiety and its persistence. These models consider three areas for intervention (threat assessment, attentional orientation, and inhibitory control) in order to develop computerized treatment that lowers attention to threat. These foster the active attentional search for both appetitive and aversive stimuli, as well as greater flexibility of inhibitory control in order to avoid developing and maintaining an anxiety disorder.

Consequently, our results suggest that the level of anxiety might have a harmful effect on the attentional and executive processes that enable the learning of emotional contingencies. However, another possible explanation comes from studies that point to these attentional and executive deficits themselves as causing anxiety and its maintenance (Mogg and Bradley, 2016, 2018; Mogg et al., 2017). In this context, the partial correlations indicate that cognitive reserve shows an indirect relation with conditioning through anxiety, enabling learning under situations of high emotional activation. It would be relevant to introduce neuropsychological tests, such as go/no-go, that evaluate executive and attentional functions as well as components of impulsivity.

Future studies that include the evaluation of attentional and executive functions would let us know whether the indirect relation between cognitive reserve and learning through anxiety, is due to the executive functions (Darby et al., 2017; Frankenmolen et al., 2018; Ihle et al., 2019; Peña-Gonzalez et al., 2020; Sauter et al., 2020). In this way, having high cognitive reserve would be associated with better executive and attentional functions, thereby involving less attentional bias, and hence, a lower level of anxiety that would permit optimal emotional learning. In this line, Ihle et al. (2019) carried out a longitudinal study where they observed that perceived stress predicted later decline in executive functions, and that this decline was mitigated in individuals over 65 who presented high scores in cognitive reserve.

In addition, it would be relevant to check whether an effect of cognitive reserve on the generalization of fear to other stimuli is observed in the high emotional group. Due to the reduced number of participants in this group, this effect could not be verified.

**FIGURE 6:**
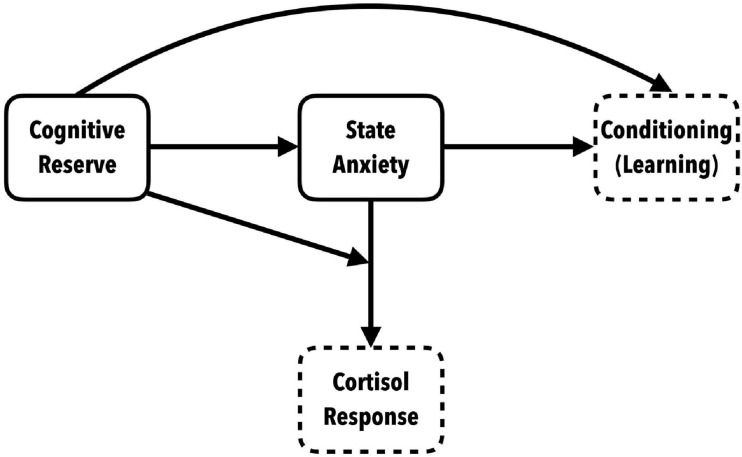
Conceptual model of the role of cognitive reserve in anxiety-triggering situations: indirect relation with conditioning and effect of interaction with SA on the neuroendocrine response.

The most clinically relevant outcome is associated with cortisol responses. Differential cortisol secretion was observed between those with high and low anxiety. The high anxiety participants showed statistically significant, higher peak cortisol levels. But high scores in cognitive reserve seems to have a protective effect on cortisol levels, especially in those who showed high state anxiety. Moreover, participants with high state anxiety and low cognitive reserve showed a higher cortisol response, along with higher maintained concentrations of cortisol after a recovery period. This indicates that having high cognitive reserve has a protective effect that mitigates reactivity to stress by significantly decreasing the phasic cortisol response in coping situations, especially in people who manifest high anxiety, and this is of vital clinical importance (see Figure 6).

These results have great clinical applicability. An intervention to improve cognitive reserve involves a comprehensive approach to introduce stimulating activities, both physical and cognitive, in a real context with high ecological validity that ensures direct learning in situations of daily life.

In conclusion, cognitive reserve shows a protective effect that mitigates the neuroendocrine response of cortisol secretion in coping situations. This is vital to prevent chronicity of high systemic levels of cortisol that increase our allostatic load, making us more vulnerable to the development of various pathologies. Cognitive reserve can therefore be considered a factor of resilience (Russo et al., 2012) to be reinforced for combating the damaging effects of stress on brain plasticity and modification of its architecture and function (Bloss et al., 2010; Holzel et al., 2010; Liston and Gan, 2011; McEwen et al., 2015b).

Thus, cognitive reserve could be an intervention strategy that could decrease the allostatic load in a sustained way. This, in turn, seems to promote learning when reported state anxiety is high (Liston et al., 2013), and to reduce the probability of pathologies developing in vulnerable individuals.

Finally, we would like to highlight some limitations of our study. Firstly, our sample is small, consists only of middle-aged men, and does not include women due to the influence of gender on endocrine stress responses. Secondly, we have only employed one stress induction paradigm. In this case, the observational fear conditioning protocol is not a stress induction paradigm but a contingency learning paradigm. However, as indicated above, this paradigm includes elements with the capacity to induce stress responses. Therefore, the protective role of cognitive reserve on cortisol responses needs to be tested in other stress induction paradigms that include high-demand mental activities and/or elements of evaluative social pressure. Third, the indirect relationship of cognitive reserve on the relationship between anxiety and learning cannot be further explored with the current data. A study involving the assessment of executive and attentional functions of low-anxious and high-anxious participants is needed to determine whether the indirect relationship of cognitive reserve on the relationship between anxiety and learning is through the relationship between cognitive reserve and executive functions.

## Data Availability Statement

The original contributions presented in the study are included in the article/supplementary material, further inquiries can be directed to the corresponding author.

## Ethics Statement

The studies involving human participants were reviewed and approved by the University of Almería Bioethics Committee (UALBIO2020/026). The participants provided their written informed consent to participate in the study.

## Author Contributions

JG-M, FC-P, JG-G, and MR-T contributed to conception and design of the study and wrote the sections of the manuscript. JG-M organized the database, extracted the data, analyzed the cortisol sample and skin conductance responses, performed the statistical analysis, and wrote the first draft of the manuscript. FC-P and MR-T analyzed the cortisol sample and performed the statistical analysis. JG-G reviewed and performed the data analysis. All authors contributed to manuscript revision, read, and approved the submitted version.

## Conflict of Interest

The authors declare that the research was conducted in the absence of any commercial or financial relationships that could be construed as a potential conflict of interest.

## Publisher’s Note

All claims expressed in this article are solely those of the authors and do not necessarily represent those of their affiliated organizations, or those of the publisher, the editors and the reviewers. Any product that may be evaluated in this article, or claim that may be made by its manufacturer, is not guaranteed or endorsed by the publisher.
