# Corrigendum: Cognitive Reserve and Anxiety Interactions Play a Fundamental Role in the Response to the Stress

**DOI:** 10.3389/fpsyg.2021.773368

**Published:** 2021-10-12

**Authors:** Jose A. García-Moreno, Fernando Cañadas-Pérez, Juan García-García, María D. Roldan-Tapia

**Affiliations:** ^1^CERNEP Research Center, University of Almeria, Almería, Spain; ^2^CEINSAUAL Research Center, University of Almeria, Almería, Spain

**Keywords:** cognitive reserve, anxiety, learning, stress, cortisol, electrodermal activity

In the published article, there was an error in affiliation 1 as published. Instead of “Department of Psychology, Health Research Center, University of Almería, Almería, Spain,” it should be “CERNEP Research Center, University of Almeria, Almería, Spain.”

Additionally, there was an error in affiliation 2 as published. Instead of “Neuroscience Laboratory, Department of Psichobiology, University of Almería, Almería, Spain,” it should be “CEINSAUAL Research Center, University of Almeria, Almería, Spain.”

The authors apologize for these errors and state that they do not change the scientific conclusions of the article in any way. The original article has been updated.

## Publisher's Note

All claims expressed in this article are solely those of the authors and do not necessarily represent those of their affiliated organizations, or those of the publisher, the editors and the reviewers. Any product that may be evaluated in this article, or claim that may be made by its manufacturer, is not guaranteed or endorsed by the publisher.

